# Enhancing Executive Functions Through Social Interactions: Causal Evidence Using a Cross-Species Model

**DOI:** 10.3389/fpsyg.2019.02472

**Published:** 2019-11-19

**Authors:** Rosemarie E. Perry, Stephen H. Braren, Millie Rincón-Cortés, Annie N. Brandes-Aitken, Divija Chopra, Maya Opendak, Cristina M. Alberini, Regina M. Sullivan, Clancy Blair

**Affiliations:** ^1^Department of Applied Psychology, New York University, New York, NY, United States; ^2^Emotional Brain Institute, Nathan Kline Institute for Psychiatric Research, Orangeburg, NY, United States; ^3^Department of Child and Adolescent Psychiatry, New York University School of Medicine, New York, NY, United States; ^4^Center for Neural Science, New York University, New York, NY, United States; ^5^Department of Population Health, New York University School of Medicine, New York, NY, United States

**Keywords:** executive function, social competence, early-life adversity, poverty, social skills, social behavior, development, longitudinal

## Abstract

It has long been theorized that humans develop higher mental functions, such as executive functions (EFs), within the context of interpersonal interactions and social relationships. Various components of social interactions, such as interpersonal communication, perspective taking, and conforming/adhering to social rules, may create important (and perhaps even necessary) opportunities for the acquisition and continued practice of EF skills. Furthermore, positive and stable relationships facilitate the development and maintenance of EFs across the lifespan. However, experimental studies investigating the extent to which social experiences contribute causally to the development of EFs are lacking. Here, we present experimental evidence that social experiences and the acquisition of social skills influence the development of EFs. Specifically, using a rat model, we demonstrate that following exposure to early-life adversity, a socialization intervention causally improves working memory in peri-adolescence. Our findings combined with the broader literature promote the importance of cultivating social skills in support of EF development and maintenance across the lifespan. Additionally, cross-species research will provide insight into causal mechanisms by which social experiences influence cognitive development and contribute to the development of biologically sensitive interventions.

## Introduction

The cognitive control abilities that enable holding and manipulating information in mind, the flexible shifting of attention between tasks, and inhibiting impulses and responses to stimuli are critical thinking skills that assist reasoning, planning, self-regulation, and management of one’s life. These higher-order cognitive abilities—called executive functions (EFs)—develop across the lifespan and are enhanced or diminished by a variety of experiential factors, especially early in life, such as environmental stimulation or stress/adversity (Perry et al., 2018b), and even physical fitness (e.g., body mass index, physical exercise) (Verburgh et al., 2014; Blair et al., 2019). Prior studies have demonstrated that these experiential factors can be successfully leveraged as points of intervention, with EF skills improving following interventions promoting stress reduction (Zelazo and Lyons, 2012) or physical exercise (Verburgh et al., 2014). However, a lesser acknowledged factor by which EF skills may also be promoted is through interpersonal experiences. This is despite strong evidence from developmental research and longstanding theory that cognitive development occurs within the context of positive social interactions and relationships (Vygotsky, 1978; Carlson, 2009; Lewis and Carpendale, 2009; Moriguchi, 2014; van Lier and Deater-Deckard, 2016).

While EF and social development are traditionally considered to be distinct domains of development, they are increasingly understood to be functionally connected. The majority of research regarding the social origins of EFs has focused on caregiver scaffolding of EF development through social interactions with their infants (e.g., Landry et al., 2002; Bibok et al., 2009; Hughes and Ensor, 2009; Roskam et al., 2014). This large body of research provides strong support that sensitive caregiving facilitates EF development. Furthermore, increasing evidence suggests that social processes influence EF development not only in infancy, but also across later development as peers become more central in youth’s lives. For example, in preschool, engaging in pretend play with peers is associated with improved self-regulation (Lindsey and Colwell, 2003). Playful interactions with peers are also associated with EF development, including cognitive flexibility (Bateson, 2005) and inhibitory control (Peterson and Flanders, 2005). Even in adolescence and adulthood, peer problems, such as peer victimization, rejection, and social exclusion, have been associated with impaired EF skills (Baumeister et al., 2002, 2005; Holmes et al., 2016). Despite these findings, the extent to which social interactions with peers may function as causal mechanisms supporting EFs is not understood.

The attainment of appropriate social skills through social interactions with peers (in addition to caregivers) may be an important driving component of EF development. In line with this idea, we recently reported findings of a novel developmental pathway whereby social competence through EF longitudinally mediated the impact of cumulative poverty-related adversities on academic achievement across the early school years (Perry et al., 2018a). Specifically, social competence in Kindergarten through EFs at Grade 1 longitudinally mediated a negative association between early-life poverty-related cumulative risk exposure and academic skills at Grade 2. These findings are in line with a growing literature that suggests that the development of social competence may be functionally linked with the development of EFs (e.g., Riggs et al., 2006; Carlson, 2009; Lewis and Carpendale, 2009; Moriguchi, 2014; van Lier and Deater-Deckard, 2016). Additionally, these results indicate that social competence may be a key mechanism by which early-life adversity impacts EF development.

Taken together, our findings paired with a broader body of literature and longstanding theory suggest that higher-order cognitive development might be facilitated, at least in part, by targeting the improvement of social skills and social interactions with caregivers and peers. Moreover, this developmental relation may be especially important for children reared in adverse environments. Indeed, a few randomized controlled trials (RCTs) provide further support for this idea. For example, interventional school curricula, such as Tools of the Mind, have incorporated Vygotskian principles into their design by not only directly scaffolding EF development, but also incorporating social pretend play to positively impact EF development (Bodrova and Leong, 1996; Diamond et al., 2007; Blair and Raver, 2014; Sasser et al., 2017). However, looking beyond the earliest school years, there is a paucity of RCTs experimentally testing if interventions that target social processes positively impact EF development. This gap in the literature persists despite well-established evidence that EF development is protracted and remains amenable to experiential input well into adolescence and adulthood (Perry et al., 2018b). Furthermore, the field is currently limited in its understanding of the causal mechanisms by which social processes operate to influence EF development, which would ultimately inform design and implementation (e.g., developmental timing) aspects of interventions to maximize effect sizes.

These gaps in the literature are likely due to normal limitations that human developmental researchers face. While prior research, including our own, has benefited from longitudinal data to begin to understand how social processes influence EF development, most studies are based on non-experimental, correlational data which limits our inferences regarding causal relations. Furthermore, we face difficulties in readily discerning cause–effect relations between social processes and EF development due to lack of experimental control within research designs involving humans. Thus, in the present study, we expanded upon our prior human findings (Perry et al., 2018a) by leveraging a rodent model with high internal validity to experimentally test our overarching hypothesis that EF development can be enhanced by targeting the improvement of social skills through facilitated social interactions. We focus specifically on the functional interplay between social development and working memory, a core EF which involves the ability to hold in mind, manipulate, and update information in one’s memory (Diamond, 2013). Working memory can be readily assessed in rodent models by using a widely used spontaneous alternation task, which is based on the tendency of rodents to explore a prior unexplored arm of a maze, and thus requires that the rodent remember which maze arms were most frequently visited (e.g., Lalonde, 2002; Hughes, 2004; Liet et al., 2015; Kraeuter et al., 2019). Importantly, spontaneous alternation also occurs in humans and has been demonstrated as early as 18 months of age (Vecera et al., 1991). Working memory is an important component of social competence as it is essential for organizing, inhibiting, and executing behavior (Riggs et al., 2006). Indeed, working memory has been associated with the facilitation of social development (Riggs et al., 2006). Furthermore, working memory develops into young adulthood and remains malleable (especially in childhood), such that working memory skills can be influenced by training (Klingberg et al., 2002, 2005) and social experiences (Perry et al., 2018b).

Thus, we employed a rodent model of early-life scarcity–adversity, which induces atypical mother–infant interactions (Perry et al., 2019) and altered social behavior across development (Raineki et al., 2012, 2015; Rincón-Cortés and Sullivan, 2016), to experimentally test if a peer socialization intervention could improve working memory in peri-adolescence. Based upon Vygotskian theory linking cognitive development to social processes, as well as prior findings that our rodent model of early-life scarcity–adversity causes social behavior problems in later life, we hypothesized that scarcity–adversity rearing would also produce cognitive development problems, as assessed via working memory performance in peri-adolescence. We additionally sought to replicate and expand upon previous results demonstrating that early-life scarcity–adversity would cause social behavior problems in juvenile and adolescent rats (Raineki et al., 2012, 2015; Rincón-Cortés and Sullivan, 2016). Furthermore, drawing from our prior human research findings suggesting that social development influences EF development (Perry et al., 2018a), we hypothesized that socializing a scarcity–adversity reared subject with a control reared rat (via co-housing) would improve the scarcity–adversity reared subject’s social behavior and working memory performance. We tested this using a peer socialization intervention spanning from time of weaning until time of testing in peri-adolescence, a developmental period which encompasses the maturation of social behavior and is increasingly thought of as a period in which neurodevelopment is sensitive to social experiences (Sisk and Foster, 2004; Schulz et al., 2009; Wei et al., 2011; Fuhrmann et al., 2015). While this rodent model is not meant to supersede the need for future human RCTs examining the efficacy of peer socialization interventions for the improvement of EFs, it serves as a valuable tool with which we can efficiently test our research questions using a tightly controlled experimental design. Furthermore, our rodent model welcomes future experiments for the assessment of specific behavioral and neurobiological mechanisms by which social interactions influence cognitive development, which would provide valuable insight into the design of mechanism-based, developmentally sensitive, biologically informed interventions.

## Materials and Methods

### Subjects

Male and female Long Evans rats were bred and raised in a temperature (20 ± 1°C)- and light (12-h light/dark cycle)-controlled room in an animal facility to provide a controlled rearing environment for all subjects. Subjects were born on postnatal day (PN) 0 and culled to 12 pups (six males, six females) on PN1. With the exception of our scarcity–adversity reared subjects (described in methods below), animals were housed with their mother in polypropylene cages (34 × 29 × 17 cm) with *ad libitum* food (Purina LabDiet #5001) and water, as well as ample wood shavings materials for nest building. Animals were weaned from their mother at PN23 and housed with one age- and sex-matched cage mate in a polypropylene cage (34 × 29 × 17 cm) with access to ample wood shavings and *ad libitum* food (Purina LabDiet #5001) and water. Animals were tested once in peri-adolescence (PN37-47, the time immediately prior to and during the onset of puberty) and each subject was only used once, with one male and one female used per litter per experimental group. All procedures were approved by New York University and Nathan Kline Institute’s Animal Care and Use Committee, in accordance with National Institutes of Health’s guidelines for the care and use of laboratory animals.

### Procedures

#### Scarcity–Adversity Rearing

On PN8, litters were randomly assigned into scarcity–adversity or control rearing conditions. In scarcity–adversity conditions the mother was provided with insufficient wood shavings materials (100 ml) for nest building in polypropylene cages (34 × 29 × 17 cm), so that she could not build a proper nest for her pups. This procedure has previously been demonstrated to negatively disrupt mother–infant interactions (Perry et al., 2019) and increase pup corticosterone release (Raineki et al., 2010). Scarcity–adversity rearing conditions persisted from PN8-12. This procedure has been used previously by our lab and others (Roth and Sullivan, 2005; Cui et al., 2006; Raineki et al., 2010, 2012, 2015; Perry and Sullivan, 2014; Rincón-Cortés and Sullivan, 2016; Doherty et al., 2017; Walker et al., 2017).

#### Peer Housing Intervention

After weaning at PN23, animals were pair-housed in polypropylene cages (34 × 29 × 17 cm) based on matched or mismatched early-life rearing conditions. In matched housing conditions, two age- and sex- matched control reared rats were housed together, or two age- and sex-matched scarcity–adversity reared rats were housed together. In mismatched housing conditions, one control reared rat and one scarcity–adversity reared rat (age and sex matched) were housed together. For all housing conditions, animals were supplied with *ad libitum* food (Purina LabDiet #5001) and water, as well as ample wood shavings materials and a plastic tube. Peer housing conditions were maintained for at least 2 weeks, spanning from weaning at PN23 until time of testing in peri-adolescence (PN37-47).

#### Spontaneous Alternation Task

Spatial working memory was assessed using a spontaneous alternation task, which is based on the natural proclivity of rodents to sequentially alternate between arms during exploration of a T- or Y-maze (Lalonde, 2002). Subjects were tested one time only in peri-adolescence (PN37-47) using a Y-maze apparatus (76.2 × 64.8 × 18.1 cm). The apparatus was constructed with a black Plexiglas floor and walls, and a clear Plexiglas lid. The maze did not contain any visual cues, but extra-maze cues were visible from all three arms to allow spatial orientation. The subject was placed in the center of the Y-maze and allowed to freely roam the apparatus for the duration of the 8-min task. All testing occurred during the light period (ZT3-ZT7, zeitgeber time, ZT0 represents light on/ZT12 represents light off). Behavior was recorded using a video camera positioned approximately 1.5 m above the apparatus. The number and sequence of arm entries were manually scored offline by an observer blinded to experimental conditions. Spontaneous alternation consists of sequential entry into each of the three arms. Therefore, percentage of spontaneous alternations was calculated by dividing the total number of alternations by the number of possible alternations: [number of alternations/(number of total arm entries − 2)]^∗^100. Through continuous assessment of spontaneous alternation, this task provides the advantage of allowing the experimenter to avoid repetitive stressful handling of subjects, such as occurs in trial-based assessments of working memory. Furthermore, this spontaneous alternation task allows for the measure of locomotor activity, as indicated by the frequency of arm entries (Hughes, 2004).

#### Social Behavior Task

Social behavior was assessed using a two-chamber Plexiglas apparatus (45.5 × 30.5 × 45 cm). The chambers were divided by a Plexiglas division with a square opening (8 × 6 cm) that allowed animals to cross between chambers. Two metal cubes (6 × 6 × 6 cm) with 1-cm circular holes were placed in each chamber. The subject was acclimated to the apparatus for 5 min prior to the start of testing. Animals were excluded from testing if they did not habituate to both chambers (spent less than 20% of time in either chamber). This exclusion criterion led to the exclusion of one control reared rat (in matched post-weaning housing) when tested in peri-adolescence. Following the acclimation period, a younger (PN25-35), same-sex animal was placed inside of the metal cube in the social stimulus chamber, while the metal cube of the other chamber remained empty. The test subject was then placed in the chamber without the social stimulus and allowed to freely roam the apparatus for the duration of the 10-min task. All testing occurred during the light period (ZT3-ZT7, zeitgeber time, ZT0 represents light on/ZT12 represents light off). Testing was recorded using Ethovision software (Noldus, Leesburg, VA, United States). Social behavior was quantified as the total time spent in each chamber, with decreased time spent in the chamber containing the social stimulus relative to the non-social chamber defined as social avoidance (Toth and Neumann, 2013). Number of crossings between chambers was also measured as an index of general locomotor activity (Raineki et al., 2012; Rincón-Cortés and Sullivan, 2016). All behavior was manually scored from videos by an observer blinded to the experimental conditions. Subjects were tested one time only in a social behavior task at either pre-weaning (juvenile; PN20-22) or peri-adolescence (PN37-47), to assess social behavior at ages immediately preceding and following the peer socialization intervention which spanned from PN23 until time of testing in peri-adolescence (PN37-47).

### Statistical Analysis

**FIGURE 1:**
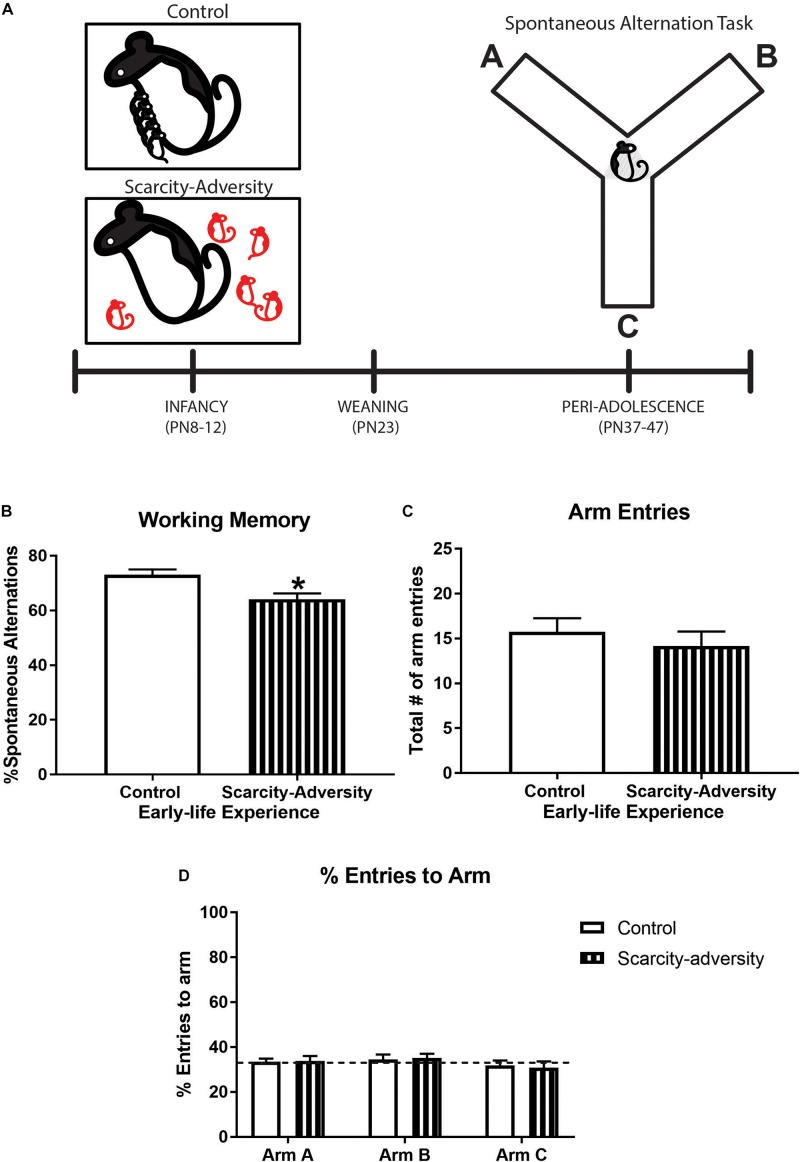
Early-life scarcity–adversity rearing reduced spatial working memory in peri-adolescence. **(A)** Experimental timeline. **(B)** Mean (± SEM) levels of percent spontaneous alternation in the spontaneous alternation task (^∗^significant difference between groups, *p* < 0.05, *n* = 7–8/group). **(C)** Mean (± SEM) levels of total number of maze arm entries during spontaneous alternation task (*n* = 7–8/group). **(D)** Mean (± SEM) percent levels of entries into each individual arm of the Y-maze during the spontaneous alternation task (dotted line represents level of entries at chance, i.e., 33%; *n* = 7–8/group).

**FIGURE 2:**
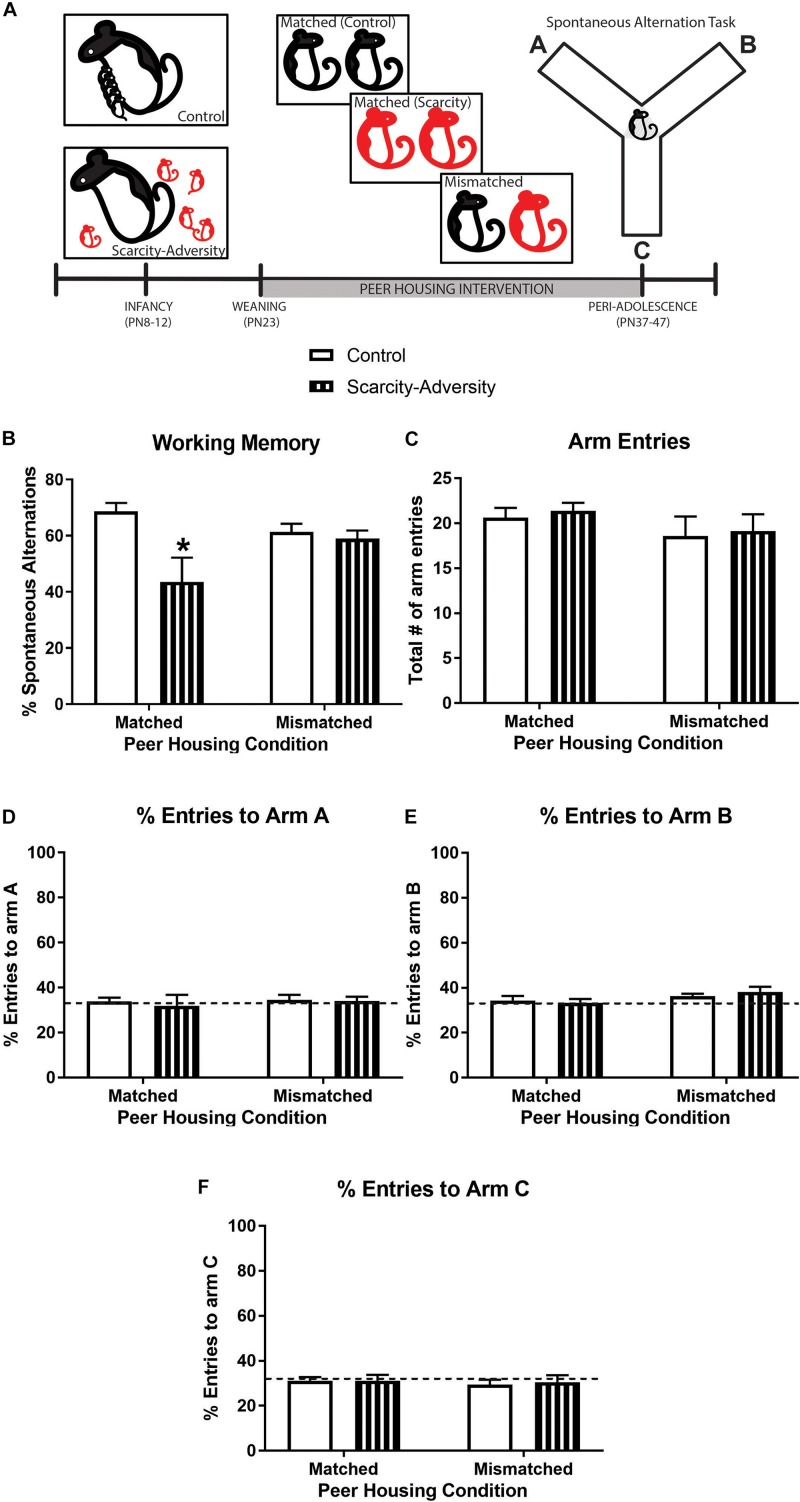
A peer housing intervention rescued spatial working memory following early-life scarcity–adversity exposure. **(A)** Experimental timeline. **(B)** Mean (± SEM) levels of percent spontaneous alternation in the spontaneous alternation task (^∗^significantly different from all groups, *p* < 0.05, *n* = 7–8/group). **(C)** Mean (± SEM) levels of total number of maze arm entries during spontaneous alternation task (*n* = 7–8/group). **(D)** Mean (± SEM) percent levels of entries into arm A of the Y-maze during the spontaneous alternation task (dotted line represents level of entries at 33%; *n* = 7–8/group). **(E)** Mean (± SEM) percent levels of entries into arm B of the Y-maze during the spontaneous alternation task (dotted line represents level of entries at 33%; *n* = 7–8/group). **(F)** Mean (± SEM) percent levels of entries into arm C of the Y-maze during the spontaneous alternation task (dotted line represents level of entries at chance, i.e., 33%; *n* = 7–8/group).

**FIGURE 3:**
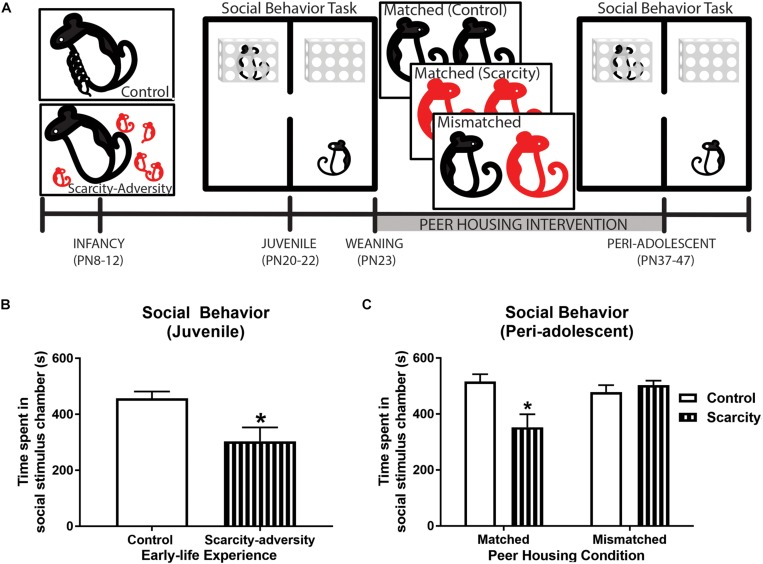
Scarcity–adversity reared subjects had improved social behavior following the peer housing intervention. **(A)** Experimental timeline. **(B)** Mean (± SEM) time spent in the social stimulus chamber during the social behavior test (^∗^significant difference between groups, *p* < 0.05, *n* = 8/group). **(C)** Mean (± SEM) time spent in the social stimulus chamber during the social behavior test (^∗^significant difference between groups, *p* < 0.05, *n* = 8/group).

All experimental data were analyzed using Prism 7 (GraphPad Software, Inc., San Diego, CA) using two-tailed Student’s *t-*tests for paired comparisons or two-way ANOVA, followed by *post hoc* Fisher’s LSD tests between groups. Significance of results was accepted at *p* < 0.05. Tests were designed assuming normal distribution and variance for control versus scarcity–adversity groups. *A priori* power analyses using G^∗^Power 3.1 software indicated that a minimum final group size of six to eight rats was required to have a probability of detecting significant group effects, depending on the experiment. Specifically, power calculation of *t*-tests comparing early-life experience indicated that a minimum sample size of six (Figure 1B) or eight (Figure 3B) rats per group was necessary to achieve power of 0.8 and an error probability of 0.05. Similar power analysis calculated the requirement of a minimum sample size of six rats per experimental group for two-way ANOVA to achieve power of 0.8 and an error probability of 0.05 (Figures 2B, 3C). All data were checked for statistical outliers using Grubbs’ outlier test. One significant outlier was removed from the control reared, mismatched housing condition for Figure 3C. Final sample sizes are as follows: Figure 1B—8 control, 7 scarcity–adversity; Figure 2B—8 control matched, 8 scarcity–adversity, 7 control mismatched, 7 scarcity–adversity mismatched; Figure 3B—8 control, 8 scarcity–adversity; Figure 3C—8 control matched, 8 scarcity–adversity matched, 8 control mismatched, 8 scarcity–adversity mismatched.

## Results

Our rodent model of early-life scarcity–adversity exposure significantly reduced peri-adolescent (PN37-47) subjects’ spatial working memory as assessed via spontaneous alternations in a Y-maze (Figure 1A), relative to control reared subjects [Figure 1B; *t*_(__13__)_ = 3.10, *p* = 0.01, Cohen’s *d* = 1.602, *t-*test]. Sensitivity analyses revealed that group differences in spontaneous alternations were not driven by differences in locomotor activity, as assessed via overall number of arm entries during the task [Figure 1C, *t*_(__13__)_ = 1.02, *p* = 0.33, *t-*test]. Additionally, *t-*tests indicated there were no group differences in percentage of entries into arm A [Figure 1D, *t*_(__13__)_ = 0.15, *p* = 0.89], arm B [Figure 1D, *t*_(__13__)_ = 0.25, *p* = 0.81], or arm C [Figure 1D, *t*_(__13__)_ = 0.31, *p* = 0.76] of the maze. Finally, one-sample *t-*tests comparing mean percent arm entries to chance performance (33% entry per arm) indicated that neither experimental group displayed a preference in entering arm A [Figure 1D, Control—*t*_(__7__)_ = 0.41, *p* = 0.69, Scarcity–adversity—*t*_(__6__)_ = 0.42, *p* = 0.69], arm B [Figure 1D, Control—*t*_(__7__)_ = 0.72, *p* = 0.50, Scarcity–adversity—*t*_(__6__)_ = 1.25, *p* = 0.26], or arm C [Figure 1D, Control—*t*_(__7__)_ = 0.50, *p* = 0.63, Scarcity–adversity—*t*_(__6__)_ = 0.77, *p* = 0.47].

However, if scarcity–adversity exposed subjects were housed with a control reared peer from weaning (PN23) until time of testing in peri-adolescence (PN37-47) (Figure 2A), the negative effect of early-life scarcity–adversity rearing on later-life working memory was attenuated. Specifically, results of a 2 × 2 ANOVA revealed a significant interaction of early-life experience (scarcity–adversity vs. control) and peer housing condition (matched vs. mismatched) on percentage of spontaneous alternations in a Y-maze [Figure 2B; *F*_(__1_,_26__)_ = 4.67, *p* = 0.04]. *Post hoc* tests indicated that scarcity–adversity subjects placed in mismatched peer housing conditions were significantly improved in percentage of spontaneous alternations relative to scarcity–adversity subjects in matched peer housing conditions (*p* < 0.05, Cohen’s *d* = 0.85). Furthermore, scarcity–adversity subjects placed in mismatched peer housing did not significantly differ from control reared subjects in their levels of spontaneous alternation (*post hoc tests*, *p* < 0.05). Sensitivity analyses indicated that group differences in spontaneous alternations were not driven by differences in overall number of arm entries during the task [Figure 2C, interaction—*F*_(__1_,_26__)_ = 0.01, *p* = 0.95; main effect of peer housing—*F*_(__1_,_26__)_ = 1.94, *p* = 0.18, main effect of early-life experience—*F*_(__1_,_26__)_ = 0.18, *p* = 0.67, 2 × 2 ANOVA]. Furthermore, 2 × 2 ANOVAs revealed that there were no group differences in percentage of entries to arm A [Figure 2D, interaction—*F*_(__1_,_26__)_ = 0.07, *p* = 0.79; main effect of peer housing—*F*_(__1_,_26__)_ = 0.20, *p* = 0.66, main effect of early-life experience—*F*_(__1_,_26__)_ = 0.15, *p* = 0.70, 2 × 2 ANOVA], arm B [Figure 2E, interaction—*F*_(__1_,_26__)_ = 0.63, *p* = 0.43; main effect of peer housing—*F*_(__1_,_26__)_ = 3.38, *p* = 0.08, main effect of early-life experience—*F*_(__1_,_26__)_ = 0.07, *p* = 0.79, 2 × 2 ANOVA], or arm C [Figure 2F, interaction—*F*_(__1_,_26__)_ = 0.05, *p* = 0.83; main effect of peer housing—*F*_(__1_,_26__)_ = 0.24, *p* = 0.63, main effect of early-life experience—*F*_(__1_,_26__)_ = 0.04, *p* = 0.84, 2 × 2 ANOVA] of the maze. Lastly, one-sample *t-*tests comparing mean percent arm entries to chance performance (33% entry per arm) indicated that neither experimental group displayed a preference in entering arm A [Figure 2D, Control Matched—*t*_(__7__)_ = 0.59, *p* = 0.57, Scarcity-adversity Matched—*t*_(__7__)_ = 0.21, *p* = 0.84, Control Mismatched—*t*_(__6__)_ = 0.66, *p* = 0.53, Scarcity-adversity Mismatched—*t*_(__6__)_ = 0.64, *p* = 0.55], arm B [Figure 2E, Control Matched—*t*_(__7__)_ = 0.67, *p* = 0.52, Scarcity-adversity Matched—*t*_(__7__)_ = 0.25, *p* = 0.81, Control Mismatched—*t*_(__6__)_ = 0.17, *p* = 0.87, Scarcity-adversity Mismatched—*t*_(__6__)_ = 0.1.45, *p* = 0.20], or arm C [Figure 2F, Control Matched—*t*_(__7__)_ = 1.12, *p* = 0.30, Scarcity-adversity Matched—*t*_(__7__)_ = 0.69, *p* = 0.51, Control Mismatched—*t*_(__6__)_ = 1.68, *p* = 0.15, Scarcity-adversity Mismatched—*t*_(__6__)_ = 0.79, *p* = 0.46].

Lastly, we checked if our peer housing mismatched condition improved scarcity–adversity reared subjects’ social behavior, as intended (Figure 3A). Pre-weaning juvenile (PN20-22) scarcity–adversity reared subjects displayed a significant reduction in time spent with a social stimulus rat during the social behavior task relative to control reared subjects [Figure 3B; *t*_(__14__)_ = 2.78, *p* = 0.015, Cohen’s *d* = 1.39, *t-*test]. Furthermore, assessment of social behavior in peri-adolescence (following the peer housing intervention) revealed a significant interaction of early-life experience and post-weaning housing condition on time spent with a social stimulus [Figure 3C; *F*_(__1_,_28__)_ = 9.49, *p* = 0.01, 2 × 2 ANOVA]. Specifically, if scarcity–adversity reared subjects were housed in matched peer housing conditions, they displayed reduced time spent with a social stimulus rat relative to control reared subjects (*post hoc tests*, *p* < 0.05, Cohen’s *d* = 1.53). However, if scarcity–adversity reared subjects were housed in mismatched peer housing conditions, they did not differ from control subjects in time spent with a social stimulus rat (*post hoc* tests, *p* < 0.05), and spent significantly more time with a social stimulus relative to scarcity–adversity subjects in matched peer housing conditions (*post hoc* tests, *p* < 0.05, Cohen’s *d* = 1.52).

## Discussion

It has long been theorized that humans develop higher mental functions, such as EFs, within the context of interpersonal interactions and social relationships (Vygotsky, 1978; Carlson, 2009; Lewis and Carpendale, 2009; Moriguchi, 2014; van Lier and Deater-Deckard, 2016). In the present study, we began to test the causal relations between social and EF development by using a rodent model to experimentally examine the contributions of peer socialization (the mismatched housing condition) to the development of working memory. Given the lack of research examining social contributions to EF development beyond early childhood, we focused our assessment on the contributions of post-weaning peer socialization on subsequent working memory performance. Specifically, we demonstrated that early-life scarcity–adversity, as modeled by rearing infant rat pups and their mother with insufficient wood shavings materials for nest building, reduced spatial working memory in peri-adolescence, as evidenced by reduced spontaneous alternation between arms of Y-maze. Notably, early-life scarcity–adversity did not produce alterations in overall number of arm entries during the Y-maze task, nor did it lead to a preference for entering a specific arm of the maze. Thus, it appears that early-life scarcity–adversity uniquely impacted spontaneous alternation between the maze arms, which we interpret as decreased working memory ability. However, we also found causal evidence that housing a scarcity–adversity reared rat with a control reared rat normalized working memory performance of scarcity–adversity reared peri-adolescents. This mismatched co-housing condition appears to have operated, at least in part, by improving scarcity–adversity reared subjects’ social behavior, which is consistent with a broad literature supporting that EFs (such as working memory) develop through social interactions and the attainment of appropriate social skills (Vygotsky, 1978; Carlson, 2009; Lewis and Carpendale, 2009; Moriguchi, 2014; van Lier and Deater-Deckard, 2016; Perry et al., 2018b).

Prior research from Sullivan and colleagues established that our early-life scarcity–adversity model induces social avoidance in juvenile, adolescent, and adult rats (Raineki et al., 2012, 2015; Rincón-Cortés and Sullivan, 2016). Here, we replicated and expanded upon these findings by providing novel evidence that this socially avoidant phenotype co-occurs with working memory problems in peri-adolescent rats. Thus, the present study’s findings supported our hypothesis that scarcity–adversity rearing would produce cognitive development problems, as evidenced via spatial working memory performance in a Y-maze. Our findings that social behavior and cognitive problems co-occurred by peri-adolescence align with increasing evidence that social and cognitive aspects of development are functionally and reciprocally linked (Riggs et al., 2006; Carlson, 2009; Lewis and Carpendale, 2009; Moriguchi, 2014; van Lier and Deater-Deckard, 2016; Perry et al., 2018b). Furthermore, our findings of scarcity–adversity induced working memory problems are consistent with human literature suggesting that poverty-related adversity negatively impacts EF development (Raver et al., 2013; Ursache et al., 2015; Perry et al., 2018b). Thus, our rodent model of scarcity–adversity appears to be somewhat translationally valid and can be further leveraged to discern behavioral and neurobiological mechanisms by which scarcity–adversity exposure influences EF development.

The present study’s findings also support our hypothesis that socializing a scarcity–adversity reared subject with a control reared rat (via co-housing) would improve the scarcity–adversity reared subject’s social behavior and working memory. Specifically, we demonstrated that pair housing a scarcity–adversity reared rat with a control reared rat rescued their socially avoidant behavior, as well as spatial working memory in a Y-maze. It is important to note that in our mismatched housing condition, the scarcity–adversity rat was not detrimental to control subjects’ social behavior or working memory post-intervention. Altogether, these findings are consistent with ours and others’ prior human research findings that support a theory of change whereby EFs of at-risk children can be improved by peer-based socialization that promotes the attainment of appropriate social skills. In humans, peers are powerful mediators of learning and gain increasing influence across development (Harris, 1995; Steinberg and Monahan, 2007; Rubin et al., 2011; Cappella et al., 2013; Telzer et al., 2018). Thus, peer-based interventions, particularly in middle childhood and beyond when peers become more central in youth’s lives, are of high potential merit for the improvement of child EF outcomes. In human research, individual peer-based socialization interventions have been successfully employed in school-based settings for the improvement of prosocial behavior (Zhang and Wheeler, 2011), externalizing or internalizing problems (Fantuzzo et al., 2005), and learning outcomes (Odom and Strain, 1984; Topping, 1996; Fuchs et al., 2008, 2009). Furthermore, peer-based interventions have leveraged natural opportunities for peer interactions in school settings to successfully overcome high student-to-staff ratios and teacher burden (Bouffard and Little, 2003; Fantuzzo et al., 2005). However, few studies have begun to assess the efficacy of peer-based interventions in improving EFs (Christ et al., 2017).

### Limitations and Future Directions

A major strength of this study was the use of an experimental design that provides high internal validity, allowing for a clearer definition of cause–effect relationships between social experiences and working memory performance. However, the current findings should be interpreted with the following limitations in mind. First and foremost, the high internal validity of the present study’s rodent experimental design comes with a trade-off to the study design’s external validity. Rodent models cannot encompass the complexity of human conditions (such as social and cultural phenomena), and thus appropriate caution should be taken when interpreting the present study’s results (Perry et al., 2019). Additionally, while the present study’s rodent findings provide causal support for the notion that peer interactions can be leveraged for the improvement of working memory, we have assessed working memory via only one outcome measure. Expanding ways in which working memory (and other measures of EFs) is assessed in rodent experimental designs would strengthen the present study’s findings and interpretations.

Indeed, future rodent research should replicate and expand upon the assessment of working memory by exploring if early adversity similarly impacts in other domains of EF development (e.g., cognitive flexibility, inhibitory control). While considered functionally distinct “core” domains of EF, working memory, cognitive flexibility, and inhibitory control are related and typically operate together (Miyake et al., 2000; Friedman and Miyake, 2017). For example, working memory and inhibitory control largely support one another such that one skill is rarely called upon without the other (Diamond, 2013). Furthermore, cognitive flexibility, which develops later, builds upon working memory and inhibitory control (Diamond, 2013). Thus, it is plausible that scarcity–adversity induced differences in working memory might co-occur with problems related to inhibitory control and cognitive flexibility. However, working memory, inhibitory control, and cognitive flexibility differ in their developmental trajectories, and are subserved by overlapping but unique neural networks (for review see Perry et al., 2018a) which could be differentially impacted by scarcity–adversity. Thus, it is also plausible that scarcity–adversity might uniquely impact the development of each core EF based on the developmental timing of adversity exposure and/or the mechanisms by which adversity influences the developing brain areas underlying EF development.

The high internal validity of our rodent model also warrants future rodent research to disentangle the mechanisms mediating the functional interplay between social processes and EF development. Indeed, future experiments should attempt to discern the specific mechanisms by which mismatched peer housing conditions improve working memory performance. For example, observations of naturalistic rodent behaviors in the mismatched housing conditions will provide evidence to if and how control reared subjects scaffold scarcity–adversity reared subjects’ social behavior. It is also possible that benefits of mismatched housing conditions are imparted via less directly observable mechanisms. For example, prior research has identified that microbial reconstitution rescues social behavior deficits in a mouse model of autism spectrum disorder (Buffington et al., 2016). Specifically, Buffington et al. (2016) utilized a mismatched housing intervention whereby offspring of mothers on a high-fat diet (MHFD) were co-housed with offspring of mothers on a regular diet (MRD). These mismatched housing conditions rescued social behavior deficits of MHFD offspring via a mechanism dependent on gut microbiota transfer from MRD offspring to MHFD offspring. Given the impact of gut microbiota on the brain (Cryan and Dinan, 2012), microbial transfer could underlie recovery of both social behavior and working memory. Future experimentation will help discern if similar mechanisms underlie our mismatched housing intervention, and thus provide important insight into means by which to improve EF development. Our rodent model can also be leveraged to determine how benefits to EF outcomes vary as a function of the developmental timing of our mismatched peer intervention, which would provide important insight for peer-based intervention efforts.

## Conclusion

In conclusion, the present study has provided novel, causal evidence that a peer-based intervention spanning from immediately post-weaning to peri-adolescence rescues early-life scarcity-adversity induced working memory problems in rodents. Furthermore, the positive effects of this peer-based intervention appear to be operating, at least in part, via the improvement of scarcity-adversity reared subjects’ social behavior. These findings converge with our lab’s previous human research, as well as prior literature supporting an overarching theory that humans develop higher mental functions such as EFs within the context of interpersonal interactions and social relationships.

To the best of our knowledge, the present findings are the first of its kind using a rodent model, which opens opportunities for studies to assess the specific behavioral and neurobiological mechanisms by which social interactions influence cognitive development. Animal models, when carefully designed and considered within the context of human research findings, provide powerful means for the efficient assessment of theory-based mechanisms of change. Furthermore, animal models have a high potential to contribute to the development of mechanism-based, biologically sensitive interventions. While EF training can be effective in many forms, interventions targeting the improvement of social skills and social interactions may prove to be particularly efficacious and generalizable across context and areas of functioning, and thus should be the focus of continued research.

## DATA AVAILABILITY STATEMENT

The datasets generated for this study are available on request to the corresponding author.

## ETHICS STATEMENT

The animal study was reviewed and approved by New York University and Nathan Kline Institute’s Animal Care and Use Committee.

## Author Contributions

RP, RS, MO, CA, and CB contributed to the conception and design of the study. RP performed rodent experiments and statistical analyses, and wrote the first draft of the manuscript. SB, MR-C, and DC performed rodent experiments. DC created illustrations for the figures. AB-A helped write sections of the manuscript. All authors contributed to manuscript revision, and read and approved the submitted version.

## Conflict of Interest

The authors declare that the research was conducted in the absence of any commercial or financial relationships that could be construed as a potential conflict of interest.
